# Postbiotics Combination Synergises the Antiproliferative Effects of Doxorubicin in Gastric Cancer Cells: A Cellular and Molecular Deep Dive

**DOI:** 10.3390/ijms27010362

**Published:** 2025-12-29

**Authors:** Radwa A. Eladwy, Mohamed Fares, Muhammad A. Alsherbiny, Dennis Chang, Chun-Guang Li, Deep Jyoti Bhuyan

**Affiliations:** 1 NICM Health Research Institute, Western Sydney University, Penrith, NSW 2751, Australia; d.chang@westernsydney.edu.au (D.C.); c.li@westernsydney.edu.au (C.-G.L.); 2 Department of Pharmacology, Faculty of Pharmacy, Egyptian Russian University, Badr City 11829, Egypt; 3 Sydney Pharmacy School, The University of Sydney, Sydney, NSW 2006, Australia; mohamed.metwaly@sydney.edu.au; 4 Pharmacognosy Department, Faculty of Pharmacy, Cairo University, Cairo 11562, Egypt; m.ali@victorchang.edu.au; 5 Freedman Foundation Metabolomics Facility, Victor Chang Cardiac Research Institute, Darlinghurst, NSW 2010, Australia; 6 School of Science, Western Sydney University, Penrith, NSW 2751, Australia

**Keywords:** postbiotics, SCFAs, gastric cancer, doxorubicin, apoptosis, proteomics

## Abstract

Short-chain fatty acids (SCFAs) acetate, propionate, and butyrate are microbial metabolites with recognised roles in gut and immune homeostasis, but their therapeutic relevance in gastric cancer, particularly in combination with chemotherapeutics, remains unclear. This study investigated the antiproliferative synergy between a combined SCFA mixture (APB) and doxorubicin (Dox) in AGS gastric adenocarcinoma cells using integrated cellular, molecular, and proteomic approaches. APB and Dox each inhibited cell proliferation, with IC_50_ values of 568.33 ± 82.56 μg/mL and 0.22 ± 0.04 μg/mL, respectively, and their combination (3000 + 0.27 μg/mL) enhanced cytotoxicity, achieving 103.46% inhibition and reducing the APB IC_50_ to 512.80 ± 18.37 μg/mL. Combination index values confirmed synergistic interactions (CI_50_ = 0.61; CI_95_ = 0.13). APB+Dox significantly increased apoptosis (94.83%) with minimal necrosis (4.64%) and induced strong ROS generation comparable to APB alone, while Dox showed limited oxidative effects. Proteomic profiling revealed downregulation of ribosomal proteins and cell cycle regulators in Dox and APB+Dox groups, with the combination further enhancing apoptosis-related pathways and stress responses. Overall, these findings indicate that SCFA-based interventions, exemplified by APB+Dox, may offer a low-toxicity strategy to potentiate chemotherapy efficacy in gastric cancer through apoptosis induction, redox disruption, and attenuation of drug resistance.

## 1. Introduction

Gastric cancer remains a major global health concern, ranked as the fifth most commonly diagnosed cancer and the fourth leading cause of cancer-related mortality as of 2020 [1,2]. The high mortality rate is largely due to late diagnosis, with many patients presenting with advanced disease [2]. Despite progress in surgical techniques, recurrence following curative resection remains common, necessitating effective adjuvant treatment strategies [3]. Chemotherapy plays a central role in the management of advanced or metastatic gastric cancer, either as neoadjuvant, adjuvant, or primary treatment [4]. Doxorubicin (Dox), an anthracycline antibiotic, has long been used in combination chemotherapy regimens for gastric cancer [5,6,7]. However, its clinical use is hampered by issues such as drug resistance and severe side effects, including cardiotoxicity, myelosuppression, and mucositis [8,9,10]. Resistance to conventional chemotherapeutics like cisplatin and 5-fluorouracil (5-FU) has further complicated treatment, often leading to suboptimal clinical outcomes [11]. Therefore, there is an urgent need to identify low-toxicity compounds with strong anticancer potential that could be integrated into new combination regimens to circumvent or mitigate drug resistance.

Diet plays a fundamental role in shaping the gut microbiota and, consequently, in maintaining host health and metabolic homeostasis [12]. Diets rich in whole grains, legumes, fruits, and vegetables provide non-digestible carbohydrates (prebiotics) that reach the colon, where they are fermented by beneficial gut bacteria into health-promoting metabolites, including short-chain fatty acids (SCFAs) [13]. Well-known fibre-fermenting bacteria such as *Faecalibacterium prausnitzii* and *Eubacterium rectale* produce SCFAs like acetate (A), propionate (P), and butyrate (B), which help maintain gut barrier integrity, modulate immune responses, and exhibit systemic anti-inflammatory effects [13,14,15]. In contrast, Westernised diets low in fibre and high in saturated fats can reduce SCFA production by disrupting the balance of these beneficial microbial species, diminishing their protective effects and contributing to chronic disease risk [12].

The gut microbiota contributes significantly to nutrient metabolism, immune modulation, and protection against pathogens [16]. Beyond maintaining gut health, increasing evidence highlights its critical role as a modulator in cancer prevention and therapy [17]. A key mechanism underlying these protective effects is the production of postbiotics, including SCFAs such as acetate, propionate, and butyrate [18,19]. These SCFAs exert both local and systemic actions relevant to cancer prevention and treatment by influencing cellular proliferation, apoptosis, oxidative stress, and immune pathways [16,17]. SCFAs are primarily generated in the large intestine through microbial fermentation of dietary fibres and are absorbed into systemic circulation, where they can exert effects beyond the gut [20]. Their intestinal concentrations vary along the colon, generally ranging from 70–140 mM in the proximal region to 20–70 mM distally, with total SCFA levels in the colon typically falling between 20 and 140 mM [20]. Butyrate, in particular, is well documented for its antiproliferative and proapoptotic properties in various cancers through mechanisms such as histone deacetylase (HDAC) inhibition, reactive oxygen species (ROS) modulation, and regulation of apoptotic signalling pathways [21,22]. Propionate and acetate have also demonstrated distinct anticancer activities, including modulation of the NF-κB and Wnt/β-catenin pathways and interference with cancer cell metabolism [23,24].

Sodium butyrate has demonstrated the ability to enhance the effectiveness of various chemotherapeutic agents [25,26,27,28,29,30]. It has been reported to sensitize tumour cells to docetaxel and, when used alongside cisplatin, significantly increases apoptosis in gastric cancer cells through activation of the mitochondrial apoptotic pathway [25,26]. B also improves the impact of 5-FU in colon cancer cells by further disrupting DNA synthesis [27]. In lung cancer, B administered prior to paclitaxel treatment helped restore gut microbiota balance, food intake, and intestinal barrier function, thereby reducing treatment-related side effects [28]. In bladder cancer, co-treatment with B and cisplatin showed synergistic anticancer effects by promoting G1-phase cell cycle arrest and apoptosis through the regulation of proteins such as p21, p27, TRADD, and procaspase-2 [29]. Additionally, propionate has been shown to enhance cisplatin’s cytotoxicity in liver cancer cells by modulating GPR41 signalling pathways [30].

In a study conducted in our lab, B synergised with dexamethasone to enhance antiproliferative effects against AGS gastric cancer cells [31]. A follow-up study evaluated combinations of SCFAs (AP, AB, PB, APB) and their co-treatment with Dex (APB+Dex) [32]. The APB+Dex combination exhibited strong synergistic interactions, targeting multiple tumour-promoting mechanisms, including the disruption of redox balance and the induction of apoptosis. Encouraged by these promising synergistic interactions between SCFAs and chemotherapeutic or immunotherapeutic agents, the current study further investigated the effects of APB, Dox, and their combination (APB+Dox) on AGS gastric adenocarcinoma cells. This novel combination aims to evaluate enhanced safety profile, induction of apoptosis, modulation of oxidative stress, and proteomic reprogramming, offering potential insights into more effective and safer therapeutic strategies for gastric cancer.

## 2. Results and Discussion

### 2.1. Antiproliferative Activity of SCFA Combinations, Dox, and Their Combination Against AGS Adenocarcinoma Cells

Magnesium acetate, sodium propionate, and sodium butyrate were used to prepare the APB mixture, reflecting the physiologically relevant salt forms of SCFAs present in the colonic lumen [20]. The concentration range (93.75–3000 μg/mL) captures both physiologically relevant exposures (0.8–10 mM) and supra-physiological conditions (up to ~26.5 mM total SCFAs) frequently used in mechanistic studies to model pharmacological exposure [20]. At the same time, our dosing strategy reflects established in vitro practice. Numerous cancer studies have applied SCFAs across 0.5–10 mM and reported dose-dependent antiproliferative and proapoptotic effects. For instance, butyrate and propionate have demonstrated growth inhibition in gastric and breast cancer cells, with IC_50_ values of ~1.3 mM (B) and ~4.5 mM (P), respectively, and apoptosis induction at higher millimolar concentrations [33,34]

In the current study, APB alone achieved 95.65 ± 7.90% inhibition at 3000 μg/mL (*p* < 0.05) with an IC_50_ of 568.33 ± 82.56 μg/mL. Dox alone (*p* < 0.05) produced 73.51 ± 5.16% inhibition at 0.54 μg/mL with an IC_50_ of 0.22 ± 0.04 μg/mL (Table 1). The combination of APB and Dox (*p* < 0.05) resulted in 103.46 ± 2.24% inhibition at 3000 μg/mL APB + 0.27 μg/mL Dox and an IC_50_ of 512.80 ± 18.37 μg/mL (Table 1). In normal Hs 738.St/Int human intestinal cells, Dox alone reduced viability to 38.37 ± 7.01% at 0.54 μg/mL. APB alone maintained 76.59 ± 8.56% viability, and the APB+Dox combination at 3000 μg/mL + 0.27 μg/mL achieved 64.12 ± 8.76% viability. At 1500 μg/mL APB + 0.136 μg/mL Dox, viability was 92.42 ± 10.66%, compared with 79.67 ± 8.16% for APB alone and 68.52 ± 7.51% for Dox alone. At concentrations ≤ 750 μg/mL APB, combination treatments produced viability values exceeding 100%, with a maximum of 112.90 ± 11.32% at 187.5 μg/mL APB + 0.017 μg/mL Dox (Table 1).

Notably, the improved viability of normal intestinal cells observed with the APB+Dox combination compared to Dox alone suggests a potential protective or toxicity-modulating effect of APB. This finding is consistent with previous reports indicating that SCFAs, particularly butyrate and propionate, can attenuate chemotherapy-induced toxicity by preserving mitochondrial function, reducing oxidative stress, and modulating inflammatory responses [35]. Such effects may be relevant to mitigating known dose-limiting side effects of Dox, including cardiotoxicity and gastrointestinal injury, as demonstrated in preclinical models [36].

### 2.2. Synergistic Potential of APB with Dox Against the AGS Cells

**Figure 1:**
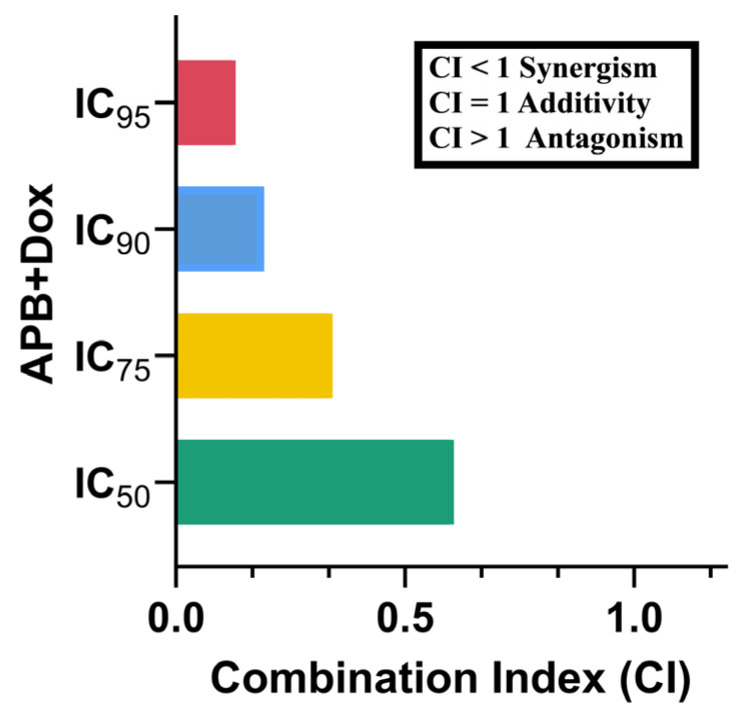
CI values of different inhibitory concentrations of APB+Dox on AGS gastric cancer cells.

The potential synergistic effects of APB and Dox combination on AGS gastric adenocarcinoma cells were analysed using the CI model [37]. The APB+Dox combination exhibited a pronounced synergistic effect, reflected in a CI value of 0.61 at the IC_50_ level, further confirming the potential of this combination to enhance therapeutic efficacy against gastric cancer (Figure 1). The IC_75_ (0.34), IC_90_ (0.19), and IC_95_ (0.13) values also showed reduced CI values, indicating that the synergy persists across a range of concentrations. These findings suggest that the inclusion of APB combinations with established chemotherapeutic agents like Dox significantly enhance the inhibitory effects on AGS gastric adenocarcinoma cells.

### 2.3. Proteomics Study of the AGS Cells Treated with the Synergistic Combination vs. Monotreatments

Following the promising cell studies and synergy studies, proteomic analysis was conducted in AGS cells after treatment with Dox (compared to the untreated control), and with the APB+Dox combination compared to Dox and APB monotherapies.

#### 2.3.1. Enrichment Analyses of Differentially Expressed Proteins (DEPs) in Dox-Treated AGS Cells Compared to the Control Untreated Cells

Dox is a widely used anthracycline chemotherapeutic that exerts its anticancer activity primarily through DNA intercalation, inhibition of topoisomerase II, and generation of ROS, which collectively lead to DNA damage and activation of apoptotic pathways [36]. The current study represents one of the first comprehensive analyses of dysregulated proteins in AGS gastric adenocarcinoma cells following treatment with Dox or its combination with APB.

**Figure 2:**
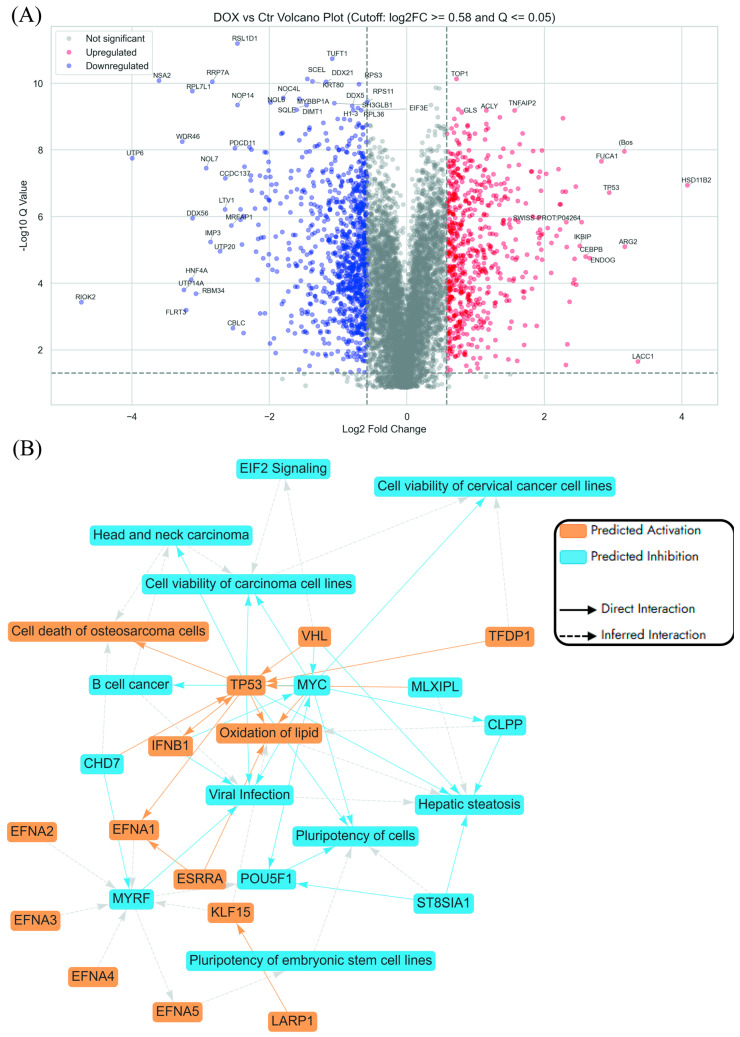
Pathway enrichment analysis using differentially expressed proteins in Dox-treated AGS cells compared to control cells. (**A**) Volcano plot showing significantly regulated proteins (absolute log2FC ≥ 0.58 and Q ≤ 0.05). (**B**) Graphical summary of top predictions in ingenuity pathway analysis (IPA) interpret report as derived from IPA core analysis.

The volcano plot of Dox treatment versus untreated control in AGS cells highlights a range of significantly dysregulated proteins, with several key players implicated in anticancer mechanisms (Figure 2A). Among the most upregulated genes is *TP53*, a pivotal tumour suppressor involved in cell cycle arrest, DNA repair, and apoptosis, whose activation reinforces Dox’s classical cytotoxic role in cancer therapy [38]. HSD11B2, also upregulated, modulates glucocorticoid metabolism and may influence stress hormone signalling within the tumour microenvironment [39]. IKBIP and CEBPB were both found to be elevated in expression, supporting their known roles in regulating apoptosis and inflammatory signalling pathways that suppress tumour survival [40]. ARG2 showed increased expression and has been associated with immune modulation and tumour progression, while ENDOG, another upregulated protein, is a mitochondrial nuclease involved in caspase-independent cell death [41,42].

On the downregulated end, several ribosome biogenesis and RNA processing genes are markedly suppressed. For example, *RIOK2*, *RPL7L1*, *RRP7A*, *NOP14*, *NSA2*, and *UTP6* are associated with ribosomal RNA synthesis and maturation [43]. Their repression may impair protein translation, contributing to reduced tumour cell proliferation. *DDX21* and *DDX56*, both DEAD-box RNA helicases, are involved in transcriptional regulation and ribosome assembly, and their downregulation may further dampen the biosynthetic capacity of gastric cancer cells [44]. Suppression of *MRFAP1*, which is linked to chromatin organisation and DNA replication, may enhance DNA damage sensitivity in the presence of Dox [45].

The graphical summary (Figure 2B) shows that Dox triggered tumour-suppressive signalling, metabolic and immune reprogramming, and inhibition of stemness-related gene expression. Key regulatory hubs such as *TP53*, *MYC*, and *TFDP1* orchestrate these changes across core biological processes, including enhanced cell death, lipid oxidation, and transcriptional regulation [38,46]. These insights suggest that Dox did more than induce apoptosis, it also reconfigured the cellular state of AGS cells, potentially weakening their proliferative and adaptive capacities and offering opportunities to overcome therapeutic resistance. A key feature of Dox’s action in AGS cells is the activation of *TP53*-mediated tumour suppression and the repression of oncogenes such as *MYC*, contributing to reduced cell viability and heightened apoptotic signalling. In parallel, modulation of lipid metabolism genes like *KLF15* and *ESRRA* points to a Dox-induced oxidative stress phenotype, known to provoke mitochondrial dysfunction and DNA damage in gastric cancer cells [47].

Dox also stimulated immune-related genes, notably *IFNB1*, reflecting activation of antiviral and inflammatory pathways that could enhance tumour immunogenicity [47]. Furthermore, the repression of stemness-associated genes such as *POU5F1* and *EFNA5* suggests an anticancer stem cell effect, with implications for limiting recurrence and resistance [48].

Among the most significantly inhibited pathways is the “Major pathway of rRNA processing in the nucleolus and cytosol”, alongside suppression of ribosomal protein synthesis and translation-associated machinery (Figure S1). These changes suggest that Dox markedly interfered with ribosome biogenesis and function, a process often hijacked in cancer to support uncontrolled protein synthesis and proliferation. In addition to ribosomal dysregulation, Dox significantly inhibited DNA-related pathways, including DNA replication, repair, and chromatin remodelling (Figure S1). These disruptions reflected Dox’s known mechanism of action as a DNA intercalator and topoisomerase II inhibitor, inducing double-strand breaks and genomic instability in cancer cells. Moreover, suppression of the cell cycle, particularly the G_2_/M checkpoint and mitotic entry, reinforced Dox’s antiproliferative impact.

Ribosomal Protein Regulation

**Figure 3:**
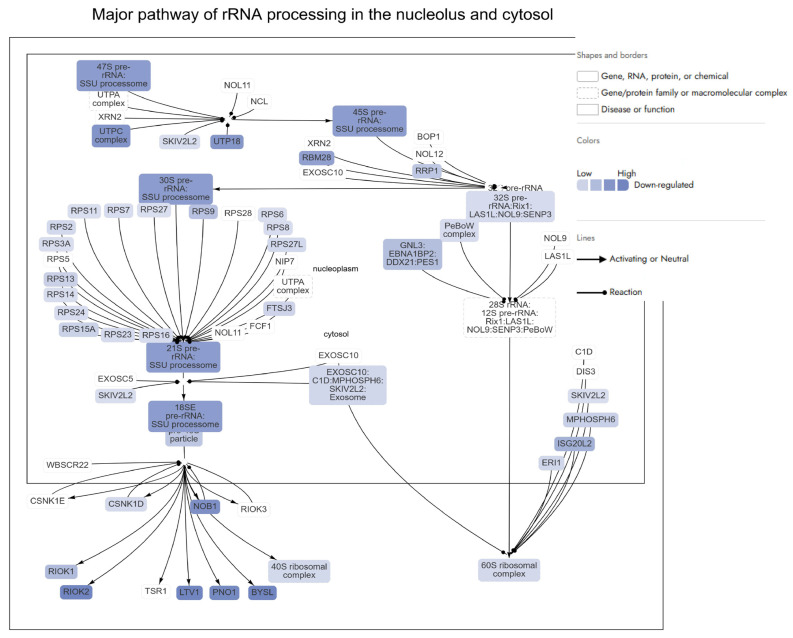
A schematic representation of the major pathway of rRNA processing in the nucleolus and cytosol. Proteins indicated in red were significantly upregulated, while proteins indicated in blue were downregulated in Dox-treated AGS cells compared to control untreated cells. The figure was generated by Qiagen Ingenuity Pathway Analysis (IPA).

Dox treatment resulted in the downregulation of multiple ribosomal proteins (RP), such as RPSA (log2FC = −0.70), RPL6 (log2FC = −0.73), RPL10 (log2FC = −0.64), RPL23 (log2FC = −0.62), RPS3A (log2FC = −0.59), RPS6 (log2FC = −0.59), RPS27 (log2FC = −0.58), RPL7A (log2FC = −0.60), RPL13A (log2FC = −1.04), RPL7L1 (log2FC = −3.12), and others (log2FC ranging from −0.58 to −3.12), which indicates that Dox suppressed ribosomal function in AGS cells [49,50,51,52,53,54] (Figure 3). These proteins are essential for various aspects of ribosome function, including protein synthesis, ribosome assembly, and translational regulation. Many of the ribosomal proteins affected by Dox, such as RPL6 (log2FC = −0.73), RPL13A (log2FC = −1.04), and RPL39 (log2FC = −0.68), have been linked to progression in gastric cancer [55,56,57]. For example, RPL6 and RPL10 (log2FC = −0.64) are associated with gastric and colorectal cancers, and RPL23 (log2FC = −0.62) is known to promote tumorigenesis [55,58,59]. The downregulation of these ribosomal proteins could be interpreted as a therapeutic effect of Dox, potentially slowing down cancer cell proliferation and invasion by interfering with the synthesis of key proteins involved in tumour progression (Figure 3). Other ribosomal proteins, such as RPS3A (log2FC = −0.59), RPS6 (log2FC = −0.59), and RPL13A (log2FC = −1.04), are not only involved in ribosome function but also have roles in cancer cell proliferation and metastasis [50,53]. Specifically, RPS6 (log2FC = −0.59), which is involved in mTOR signalling, is often activated in gastric cancer, and its downregulation by Dox might impact this critical pathway, potentially reducing cancer cell growth and metastasis [60,61].

Some of the genes affected by Dox, such as *RPS27A* (log2FC = −0.63), *RPL38* (log2FC = −0.65), and *RPL14* (log2FC = −0.77), are involved in ribosome stability and function, and their dysregulation is associated with cancer progression and metastasis [51,62,63]. Additionally, serine/threonine-protein kinase SMG enzymes were reported to decrease by a DNA damage inducer, such as Dox [64]. In the current study, genes such as SMG1 (log2FC = −0.61), SMG6 (log2FC = −0.80), and SMG7 (log2FC = −0.92), which are involved in nonsense-mediated mRNA decay (a component of the DNA repair response), show downregulation in response to Dox. This suggests that the drug may influence the cellular DNA damage response in a way that could further reduce the ability of cancer cells to proliferate and survive under stressful conditions.

The downregulation of key ribosomal proteins and associated biogenesis factors—such as RIOK2, UTP6, NSA2, and WDR46—indicates that Dox significantly impairs ribosome maturation and assembly (Figure 3) [65]. RIOK2, for example, is critical for the late-stage maturation of the 40S ribosomal subunit. Its marked downregulation (log2FC = −4.73) by Dox potentially inhibited the formation of functional ribosomes, reducing the translational capacity of cancer cells [65]. Similarly, UTP6 (log2FC = −3.99) and UTP14A (log2FC = −3.24), integral components of the small subunit processome, are involved in pre-rRNA processing [66]. Their suppression disrupts ribosomal RNA synthesis, thereby limiting ribosome production. Downregulation of UTP6 and UTP14A is associated with drug resistance and poor prognosis in gastric cancer [67,68]. Combination therapies (such as APB+Dox) targeting complementary pathways may help overcome resistance.

The downregulation of ribosomal proteins such as DDX56 (log2FC = −3.12) and RRP7A (log2FC = −2.83) underscored Dox’s broad impact on ribosome biogenesis [43,69]. These proteins are crucial for various stages of ribosomal assembly, and their inhibition by Dox suggests a comprehensive disruption of this process. The suppression of proteins like WDR46 (log2FC = −3.27) and NSA2 (log2FC = −3.61) highlighted Dox’s potential ability to induce ribosomal stress, a state that sensitizes cancer cells to apoptosis [70,71]. WDR46, a scaffold protein essential for nucleolar structure and ribosomal RNA processing, is crucial for maintaining the nucleolus, a hub of ribosome biogenesis [70]. Its downregulation disrupts nucleolar integrity, a phenomenon that has been linked to the activation of p53-mediated apoptotic pathways [72]. Similarly, the inhibition of NSA2, which facilitates 60S ribosomal subunit assembly, further compromises ribosomal function, halting protein synthesis and cancer cell growth [73].

DNA-related processes

Dox also impacted DNA-related processes in AGS gastric cancer cells, targeting key proteins involved in DNA repair, replication, and stability. Its effects were evident in multiple pathways that regulate DNA damage response (DDR), replication fork dynamics, and chromatin stability, collectively contributing to its anticancer efficacy (Figure 3). Among the DDR (DNA damage response) proteins, Poly(ADP-ribose) polymerases (PARPs) such as PARP2 (log2FC = −1.43), PARP4 (log2FC = −1.04), and PARP12 (log2FC = −0.94), all of which are central to DNA repair mechanisms, showed significant downregulation. Dox is known to impair DDR by targeting key genes, such as *PARPs*, a crucial player in DNA repair that interacts with DNA strand breaks [74]. *BRCA*-encoded protein is crucial for the homologous recombination repair mechanism, which is vital for repairing double-strand breaks caused by Dox [75]. The loss of BRCA1 expression has been shown to enhance resistance to Dox in other cancer models, where BRCA1 deficiency triggers resistance mechanisms [75]. The downregulation of *BRCA1* (log2FC = −0.80) in AGS cells treated with Dox suggests that the DNA damage response (DDR) pathway may be compromised, impairing the cells’ ability to repair DNA effectively. The suppression of these genes potentially compromised the recombination repair, a vital pathway for repairing DNA double-strand breaks. In addition, the reduction in *RAD51* (log2FC = −0.67) further hindered this repair process, leading to increased genomic instability and heightened susceptibility to apoptosis in cancer cells [76]. Interestingly, *XPC* (log2FC = 1.13), a gene involved in nucleotide excision repair, was upregulated, suggesting a cellular attempt to counteract the widespread DNA damage. This is in line with a previous study demonstrating the overexpression of *XPC* gene in Dox-treated cells [77].

Dox also impacted proteins critical for maintaining DNA metabolism and chromosomal stability. Telomeric repeat-binding factor 1 (TERF1) is a key protein within the telomere complex essential in mediating the interactions between telomeres in mammalian cells [78]. It helps regulate telomere length and stability by binding to the telomeric DNA repeats, thereby facilitating the structural integrity of the telomeres [79]. The observed downregulation of *TERF1*-encoded protein (log2FC = −1.22) suggests potential telomere destabilization, which may contribute to genomic instability. In addition, the reduced expression of replication fork-associated genes, such as *SMARCAL1* (log2FC = −0.68) and *SMARCAD1* (log2FC = −0.71), was aligned with disruptions in DNA replication fidelity, exacerbating genomic instability [80]. The significant downregulation of *SETD2* (log2FC = −2.02), responsible for histone *H3K36* methylation, may further impair chromatin remodelling and repair, weakening the cell’s ability to maintain genomic integrity [81]. *SETD2* mutations contribute to resistance to Dox by impairing the DDR, weakening the apoptotic response to chemotherapy [82]. In gastric cancer, the downregulation of proteins like MUC1 (log2FC = −0.68), AXIN2 (log2FC = −1.50), and MGMT (log2FC = −0.77) potentially indicate the disruption of DNA damage response and signalling pathways, with Dox having a broad impact on these processes [83,84,85,86]. While MGMT is more closely associated with alkylating agents, its downregulation in this context could reflect an indirect effect [85]. Additionally, the suppression of FANCG (log2FC = −1.99), a key player in the Fanconi anaemia pathway, indicates a probable disruption of DNA repair mechanisms in the AGS gastric cancer cells, contributing to increased DNA damage [87].

SOX9, a transcription factor crucial for cartilage formation and stem cell regulation, also plays roles in cellular responses to DNA damage [88]. STAT6, another transcription factor, is involved in the regulation of immune responses and inflammation, which may be critical in response to Dox-induced cellular stress [89]. The downregulation of SOX9 (log2FC = −1.33) and STAT6 (log2FC = −0.84) suggests disruptions in transcriptional regulation related to DNA repair and inflammation [88,89]. Their downregulation could impact cellular responses to DNA damage and inflammatory signals, which aligns with the observed effects of Dox treatment. Additionally, the upregulation of *SYVN1* (log2FC = 1.30), a gene involved in the cellular stress response and protein degradation, indicates an active, though insufficient, compensatory mechanism to mitigate the damage [90]. *SYVN1*’s role in responding to DNA-damage-induced stress further reflects the cell’s attempt to cope with Dox-induced damage, but the response may not be enough to overcome the widespread effects of the drug [90]. Finally, Dox modulated proteins linked to DNA replication complexes and chromatin remodelling. While GINS2 (log2FC = 1.46) and GINS4 (log2FC = 1.41) were upregulated, potentially reflecting compensatory mechanisms to sustain replication, the suppression of histone methyltransferases such as ASH1L (log2FC = −1.26) potentially limited the capacity for chromatin repair and transcription regulation [91,92].

Cell Cycle

Dox significantly impacted the cell cycle in AGS gastric cancer cells by targeting key regulatory pathways and proteins involved in cell cycle progression. From the data provided, Dox induced disruptions at multiple checkpoints, particularly within the G_1_/S and G_2_/M phases, thereby promoting cell cycle arrest.

*G*_1_/*S Phase Arrest*

Dox’s action began at the G_1_/S transition, disrupting the progression into the S phase by downregulating critical cyclins and CDKs that control the checkpoint. Key proteins affected include CCND1 (log2FC = −1.90), CDK4 (log2FC = −1.34), and CDK6 (log2FC = −0.77), leading to reduced phosphorylation of Rb protein and failure to activate E2F transcription factors [93,94]. The suppression of E2F4 (log2FC = −1.71) further inhibited the transcription of *S*-phase related proteins, compounding the block at this checkpoint (Figure 3) [95]. Moreover, the upregulation of TP53 (log2FC = 2.94) reinforced this arrest through the activation of p21 (CDKN1A), a cyclin-dependent kinase inhibitor (CDKI), which binds to and inhibits CDK4/6 activity [96]. This halted cell cycle progression and allowed cells to initiate DNA damage repair or apoptosis [96]. The overall inhibition at this stage potentially prevented AGS cells from replicating damaged DNA, a hallmark of effective anticancer action. Interestingly, despite the suppression of key G1/S drivers, the upregulation of CCND3 (log2FC = 1.03) and CCNE1 (log2FC = 1.58) suggests that some compensatory mechanisms might be activated in response to Dox-induced stress [97]. However, these attempts were insufficient to overcome the dominant inhibitory effects mediated by Rb and p53.

*S Phase Disruption*

While the G_1_/S arrest was prominent, Dox also impacted cells that entered the S phase. The downregulation of replication factors like MCM10 (log2FC = −0.80) and proteins associated with replication origin licensing disrupted DNA replication initiation [98]. The reduced expression of PCNA (log2FC = 0.68), a critical replication clamp protein, further hampered replication fork progression [99]. These disruptions created replication stress, which is exacerbated by the suppression of checkpoint kinases CHEK1 (log2FC = −1.11) and CHEK2 (log2FC = −1.07) [100]. These kinases are vital for detecting and repairing stalled replication forks [100]. Their downregulation allows replication stress to persist, accumulating DNA damage and driving cells toward apoptosis instead of completing the S phase.

*G*_2_/*M Phase Arrest*

Dox may also profoundly affect the G_2_/M checkpoint, preventing cells with damaged DNA from entering mitosis. The downregulation of PLK1 (log2FC = −0.73), a key regulator of mitotic entry and spindle assembly, disrupted mitotic initiation [101]. Similarly, the suppression of MAD2L2 (log2FC = −1.10) and BUB1 (log2FC = −0.88) interfered with spindle checkpoint signalling, leading to improper chromosomal alignment and segregation [102,103]. Separase regulators like ANAPC7 (log2FC = −0.77), along with the upregulation of PTTG1 (Securin) (log2FC = 0.90), reflected a broader disruption of chromosome segregation machinery [104,105]. Overexpression of PTTG1 in normal human fibroblasts activates the DNA damage response pathway, leading to p53-dependent cell cycle arrest [105].

*Mitotic phase*

Mitotic disruption caused by Dox culminates in mitotic phase arrest, a process characterized by cell death following defective mitosis. The *TP53* gene, which encodes the p53 protein, plays a central role in cell cycle arrest by stopping the cell cycle and preventing the spread of DNA-damaged cells [106]. Upregulation of *TP53* (log2FC = 2.94) plays a central role in this outcome by enforcing checkpoints and initiating apoptosis. Furthermore, the observed downregulation of NDC80 (log2FC = −1.08), a kinetochore complex component, potentially disrupted microtubule attachment and chromosome alignment, further impairing mitotic fidelity [107]. Proteins like ESPL1 (Separin) (log2FC = 0.81) and other regulators of chromosome segregation were affected in a way that enhanced chromosomal instability, creating a lethal environment for cancer cells [108].

#### 2.3.2. Enriched Pathways Using DEPs of APB+Dox Combination-Treated AGS Cells vs. Monotreatments

**Figure 4:**
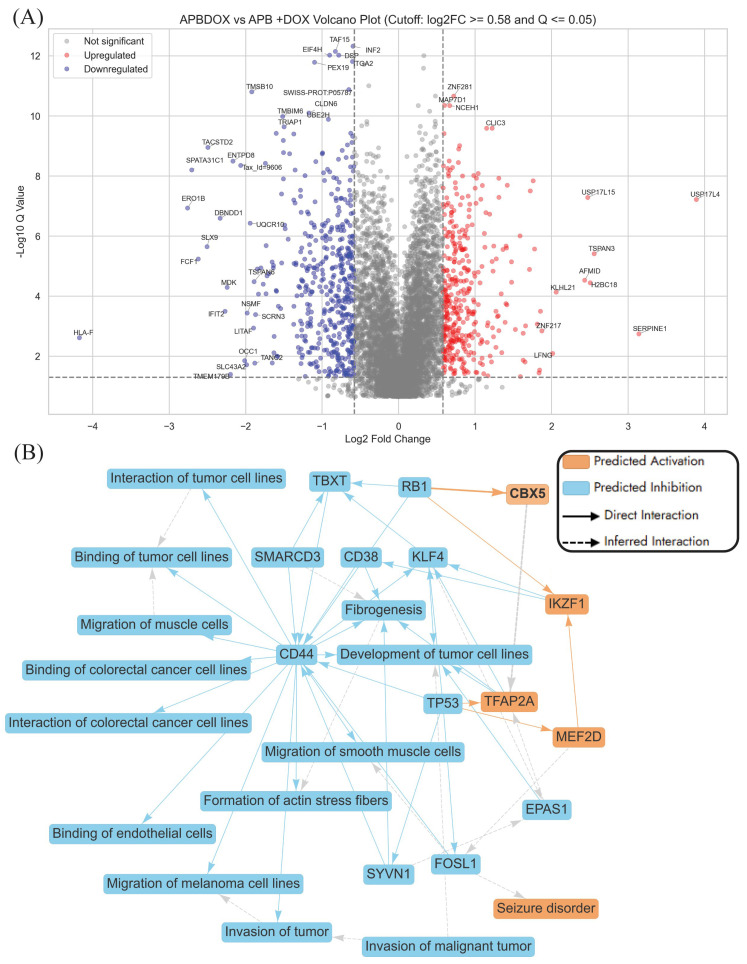
Pathway enrichment analysis using differentially expressed proteins in APB+Dox-treated AGS cells compared to APB and Dox (Q ≤ 0.05). (**A**) Volcano plot showing significantly regulated proteins (absolute log2FC ≥ 0.58 and Q ≤ 0.05). (**B**) Graphical summary of top predictions in an IPA Interpret report presented in the form of a simple network.

Figure S2 illustrates the machine-learning-based prediction of molecular effects on the malignant neoplasm of the aerodigestive tract signalling pathway, highlighting the impact of APB+Dox treatment compared to the control (Figure S2). This systems-level prediction corresponds closely with proteomic changes identified in the volcano plot (Figure 4A), which compares APB+Dox treatment to individual APB and Dox monotherapies and highlights several differentially expressed genes potentially relevant to the anticancer activity of the combination in AGS gastric adenocarcinoma cells. Notably, several downregulated genes, such as *HLA-F*, *TRIAP1*, *ENTPD8*, *TMBIM6*, and *TMSB10*, are known to play roles in cell survival, mitochondrial integrity, and antiapoptotic processes. *HLA-F*, a non-classical class I molecule, is implicated in cancer, often upregulated in various types of tumours [109,110,111]. It can be found in cancer cells, potentially allowing them to evade immune surveillance and suppress antitumour immune responses [109]. Additionally, *HLA-F* is associated with poor survival in some cancers, such as glioma and non-small-cell lung cancer [109]. *TRIAP1* is involved in mitochondrial membrane homeostasis and cell protection from apoptosis, while *TMBIM6* is associated with resistance to stress-induced cell death [110,111]. Their suppression suggests enhanced apoptotic susceptibility when APB is used in conjunction with Dox. Additionally, *ENTPD8*, which has been linked to purinergic signalling and immune evasion in tumours, is also significantly downregulated, potentially improving immunogenicity [112]. On the other hand, genes such as *SERPINE1*, *USP17L4*, and *TSPAN3* were significantly upregulated. *SERPINE1* (plasminogen activator inhibitor-1) is known to modulate extracellular matrix remodelling and may either promote or suppress tumour progression depending on context; however, in this combination, its induction may reflect a stress or reparative response to extensive damage (Figure 4A) [113]. The upregulation of deubiquitinases like *USP17L4* and *USP17L15* could be compensatory but also point to altered proteostasis under APB+Dox treatment [114].

The graphical summary of APB+Dox vs. APB and Dox monotreatment revealed a distinct suppression of tumour-promoting mechanisms in AGS gastric cancer cells, with a particular focus on *CD44*-driven signalling (Figure 4B). *CD44*, a well-established marker of cancer stem cells and a facilitator of tumour progression in gastric cancer, is central to this network [115]. It influences key processes such as tumour cell interaction, migration, invasion, and fibrogenesis [115]. Its downregulation, along with that of *CD38*, suggests a disruption in tumour–stromal interactions and extracellular matrix remodelling—hallmarks of aggressive gastric cancer phenotypes (Figure 4B) [116]. The associated suppression of fibrogenesis-related genes like *EPAS1*, *SMARCD3*, and *SYVN1* supported the potential of combination therapy to interfere with tumour microenvironment remodelling, which is critical for tumour invasion and metastasis (Figure 4B) [117]. Further, the network indicates transcriptional reprogramming as a major theme, with reduced activity in transcriptional regulators such as *KLF4*, and *EPAS1*, which have been implicated in gastric tumour growth and epithelial-to-mesenchymal transition (EMT) [118,119]. The suppression of genes like *FOSL1* and *SYVN1*, which support actin stress fibre formation and cellular motility, suggests impaired cytoskeletal dynamics and reduced metastatic capacity [90,120].

The canonical pathway analysis comparing APB+Dox to the monotreatments (APB and Dox individually) highlighted significant alterations in pathways related to cell cycle regulation, apoptosis, rRNA metabolic processes, and elements associated with invasion of tumour pathways (Figure S2). There was marked upregulation of cell cycle checkpoints, DNA double-strand break response, and mitotic progression pathways, suggesting a strong activation of mechanisms that halted the cell cycle in response to genotoxic stress. This aligns with the mode of action of Dox, which induces DNA damage, and indicates that APB+Dox may enhance this effect to inhibit cancer cell proliferation [121]. Pathways associated with apoptosis and cellular stress, including oxidative-stress-induced senescence and the senescence-associated secretory phenotype, were also upregulated, pointing to an increased likelihood of cancer cells undergoing programmed cell death or permanent arrest. Interestingly, rRNA-related processes such as RNA polymerase I transcription and regulation of rRNA expression were activated, possibly reflecting a cellular response to stress or compensatory mechanisms to maintain protein synthesis under damage conditions.

Invasion of tumour pathways

**Figure 5:**
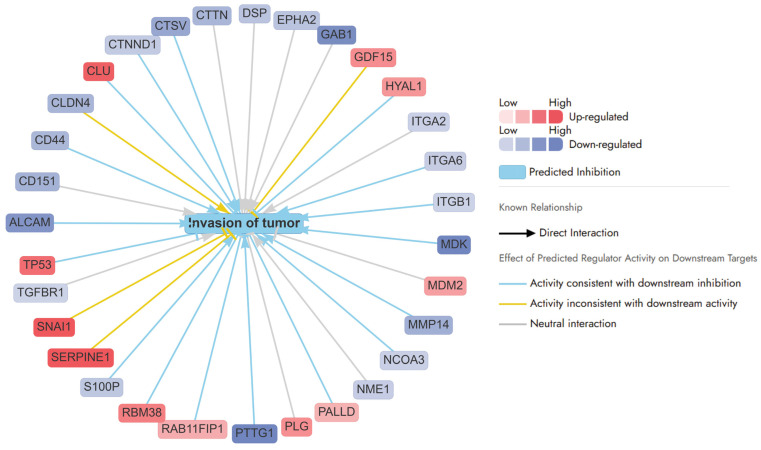
Network analysis of differentially expressed proteins involved in tumour progression following APB+Dox treatment in AGS cells compared to monotreatments APB and Dox.

The APB+Dox combination exerted potent anticancer effects in AGS gastric cancer cells by disrupting multiple pathways involved in tumour invasion, angiogenesis, and epithelial-to-mesenchymal transition (EMT). As illustrated by the network analysis, this treatment resulted in the coordinated downregulation of key genes associated with cell adhesion (e.g., *CD44* (−0.96), *CD151* (−1.03), *ALCAM* (−1.43), *CLDN4* (0.99), integrins (*ITGA2* (−0.60), *ITGA6* (−0.65), *ITGB1*(−0.59)), and extracellular matrix (ECM) remodelling proteins such as MMP14 (−1.07), CTSV (−1.18), and MDK (−2.24) (Figure 5). These changes suggest a marked impairment of tumour−stromal interactions and metastatic capacity. A particularly notable target, *MDK* (Midkine), a growth factor critical for angiogenesis and vascular remodelling, was significantly downregulated (log2FC = −2.24), indicating suppressed tumour-supporting vasculature. Similarly, *TACSTD2*, a gene linked to EMT and cancer progression, was strongly suppressed (log2FC = −2.49), likely contributing to reduced metastatic potential. Other downregulated factors such as *ATP1B1* (log2FC = −0.74), *EPCAM* (log2FC = −1.72), and *CD44* (log2FC = −0.96) further suggest disrupted cellular homeostasis, loss of cancer stemness, and inhibition of tumour recurrence (Figure 5).

Conversely, the upregulation of tumour suppressors like *TP53* (1.16) and *RBM38* (1.02), and stress regulators such as *CLU* (1.28), supports a shift towards apoptosis and reduced invasiveness. *TGFBR1* (−0.59), although typically involved in growth factor signalling, may, under this treatment context, exert anti-invasive effects. The network predominantly features predicted inhibitory interactions (blue arrows), reinforcing the hypothesis that APB+Dox disrupts invasion-related pathways through suppression of adhesion, ECM degradation, and cell signalling mechanisms. *SNAI1* (log2FC = 1.71), a key EMT transcription factor, and *SERPINE1* (log2FC = 3.14), a regulator of extracellular matrix remodelling and cell migration, were upregulated. This might suggest a partial or compensatory EMT response despite the broader anti-invasive effects of the treatment (Figure 5). Similarly, *GDF15* (log2FC = 0.91), typically linked to growth factor signalling, and *CLDN4* (log2FC = −0.99), involved in tight junction integrity, displayed expression patterns inconsistent with the predicted inhibition of tumour invasion (Figure 5). These signals may reflect a compensatory effect or stress adaptation mechanisms and highlight the complexity of interpreting network-level responses, where isolated proinvasive signals may persist within an overall suppressive theme.

Apoptosis

The APB+Dox combination exerted a potent anticancer effect on AGS adenocarcinoma cells primarily through apoptosis modulation. Apoptosis is tightly regulated through both intrinsic (mitochondrial-mediated) and extrinsic (death-receptor-mediated) pathways [122]. In AGS cells treated with the APB+Dox combination, the expression of proapoptotic and antiapoptotic factors was significantly altered, contributing to the observed therapeutic effects (Figure S4).

*Intrinsic Pathway of Apoptosis*

The intrinsic apoptotic pathway involves mitochondrial outer membrane permeabilization (MOMP), leading to cytochrome c release and caspase activation [123]. *BID* (log2FC = −0.75), a BH3-only protein that activates BAX and BAK, showed a slight downregulation [123]. Despite this, the proapoptotic *BCL2L11* (BIM, log2FC = 1.10) was upregulated, tipping the balance in favour of apoptosis by antagonizing antiapoptotic proteins like BCL-2 and BCL-XL [124]. Concurrently, *CASP9* (log2FC = −0.69), essential for apoptosome formation, was modestly downregulated, potentially modulating the extent of apoptosis [125]. Several genes influencing redox balance and mitochondrial health, such as *ERO1B* (log2FC = −2.76) and *ENDOG* (log2FC = −0.91), were downregulated. *ERO1B*’s decrease may amplify ER-stress-mediated apoptosis, while *ENDOG*’s reduction might partially offset DNA fragmentation effects [126,127]. Additionally, *ERN1* (log2FC = 1.65), associated with the unfolded protein response (UPR), was upregulated, indicating enhanced ER stress that can synergize with mitochondrial apoptosis [128].

*Extrinsic Pathway of Apoptosis*

The extrinsic pathway is impacted through the modulation of death receptor signalling [123]. *TNFRSF10B* (TRAIL receptor 2, log2FC = −1.19) and *TRADD* (log2FC = −0.67) were downregulated upon APB+Dox treatment, which could dampen direct TRAIL-mediated apoptosis [129,130]. However, TRAIL expression and its downstream signalling may still induce apoptosis in susceptible cells [129]. *CASP10* (log2FC = −0.68), a caspase involved in extrinsic pathway signalling, showed slight downregulation, but this effect may not fully abrogate extrinsic apoptosis.

*Crosstalk Between Pathways*

The APB+Dox combination facilitated crosstalk between the intrinsic and extrinsic pathways. *JUN* (log2FC = 0.60), part of the AP-1 transcription complex, was upregulated, potentially driving proapoptotic gene expression [131]. Similarly, *TP53* (log2FC = 1.16), the “guardian of the genome”, was upregulated, enhancing both intrinsic and extrinsic apoptosis by potentially inducing genes like *BAX*, *PUMA*, and *NOXA* [132]. *TP53* upregulation was also observed in the Dox-only treatment (log2FC = 2.94), where it contributed to cell cycle arrest via activation of the CDK inhibitor p21 (CDKN1A), which inhibits CDK4/6 and halts progression through the G1/S checkpoint. In the APB+Dox group, *TP53* activation may also counteract the survival-promoting influence of antiapoptotic genes such as *RAF1* (log2FC = 0.89) [133]. *TP53* acts as a central regulator of cellular fate, integrating multiple stress responses to maintain genomic integrity. As shown in Figure S4, TP53 promotes apoptosis through activation of proapoptotic genes such as *BAX*, *PMAIP1* (PUMA), *BBC3*, and *CASP6*, while simultaneously inhibiting cell survival by repressing antiapoptotic factors including *BCL2*, *BCL2L1*, and *BIRC5* [134,135]. *TP53* also induces autophagy via DRAM1 and promotes senescence through *SERPINE2*, reinforcing its role in halting the proliferation of damaged cells [136,137]. Moreover, *TP53* enhances mitochondrial respiration by activating *SCO2* (log2FC = 0.74), which supports oxidative phosphorylation, while concurrently suppressing glycolysis through upregulation of *TIGAR*, thereby shifting metabolism away from anaerobic energy production [138,139].

rRNA metabolic process

*rRNA Biogenesis and Ribosomal Genes*

Ribosome biogenesis is a hallmark of rapidly dividing cancer cells. Key genes involved in rRNA synthesis and ribosome function, including *RPL14* (log2FC = 0.59), *UBA52* (log2FC = 0.60), and *RRN3* (log2FC = 0.76), were upregulated in response to APB+Dox treatment [140,141,142]. While these proteins are generally associated with ribosome assembly, their increased expression in this context may reflect a compensatory or stress-adaptive response rather than enhanced global protein synthesis. *UBA52* has been reported to simultaneously deliver both *RPL40* and ubiquitin to the ribosome [141]. These genes play a critical role in driving ribosome assembly and protein synthesis, processes essential for tumour progression. Elevated expression of *RPL14* and *UBA52* has been linked to increased protein synthesis, metabolic adaptation, and enhanced resistance to cellular stress. Notably, higher levels of *RPL14* have also been shown to suppress cell migration and invasion in nasopharyngeal carcinoma [140]. Conversely, *RIOK2* (log2FC = −0.95), a kinase promoting ribosome assembly, was downregulated, potentially disrupting ribosome biogenesis [65]. This downregulation may inhibit tumour growth by reducing ribosomal output and impairing the protein synthesis machinery that supports cancer cell proliferation [65].

*RNA Processing and Splicing*

The APB+Dox combination influences genes involved in RNA processing and splicing, key processes in post-transcriptional gene regulation. Genes such as *SMN2* (log2FC = 0.73) and *ISY1* (log2FC = 0.69), involved in RNA splicing, were upregulated, suggesting an adaptive response to therapy [143,144]. Increased RNA splicing activity could represent an effort by cancer cells to maintain proper gene expression under therapeutic stress. In contrast, *DCAF13* (log2FC = −1.77), which regulates ubiquitination and degradation of proteins, was significantly downregulated [145]. *DCAF13*, a substrate receptor in the CUL4-DDB1 E3 ligase, is known for promoting cancer cell migration, invasion, and epithelial–mesenchymal transition [146]. Interestingly, while Dox treatment has been reported to upregulate *DCAF13*, potentially enhancing metastatic risk, SCFAs may counteract this effect by downregulating *DCAF13* (−1.77 for APB+Dox treatment), offering a therapeutic advantage in reducing metastasis during chemotherapy [146]. The APB+Dox combination exerted its anticancer effects by targeting vulnerabilities in the rRNA metabolic process. While some ribosomal and RNA processing genes are upregulated, potentially as a compensatory response, the downregulation of critical genes like *RIOK2* and *DCAF13* potentially disrupted ribosome biogenesis and protein degradation pathways. This dual modulation leads to metabolic and nucleolar stress, impairing the cancer cell’s ability to sustain rapid growth and survival.

Cell Cycle

The APB+Dox combination substantially impacted the cell cycle in AGS gastric cancer cells, disrupting essential processes related to mitosis, chromatin organization, and DNA repair. These effects collectively impair normal cell division, contributing to the anticancer efficacy of the treatment. In the current study, APB+Dox treatment led to marked upregulation of *TP53* (log2FC = 1.16) and several key checkpoint regulators, including *CHEK2* (log2FC = 0.86), *CDC20* (log2FC = 1.24), and *CDCA8* (log2FC = 0.74) [134]. It is noteworthy that the Dox treatment resulted in upregulation of *TP53* (log2FC = 2.94). Interestingly, this was accompanied by the downregulation of negative mitotic regulators such as *PKMYT1* (log2FC = −0.65) and *UBE2N* (log2FC = −1.02), suggesting a coordinated shift toward checkpoint activation rather than evasion. Notably, overexpression of *PKMYT1* and *UBE2N* has been associated with poor prognosis in breast and prostate cancers, respectively, supporting their role in tumour progression [147,148]. Additionally, the suppression of *UBE2N*, *UBE2C*, and *PSMB10* (log2FC = −1.02 to −0.72)—key components of the ubiquitin–proteasome system—indicates inhibition of proteasomal degradation, which may stabilize proapoptotic proteins and enhance cell death [147,149,150]. Other mitotic regulators such as *SGO2* and *PKMYT1*, which are crucial for chromosome cohesion and *CDK1* inhibition, were also downregulated (log2FC = −0.89 and −0.65, respectively), suggesting further interference with proper mitotic progression [151]. A notable target is *KLHL21* (Kelch-like protein 21), which was significantly upregulated (log2FC = 2.06) [152]. *KLHL21* is essential for mitotic progression and cytokinesis, ensuring accurate chromosome segregation during cell division. Its upregulation under APB+Dox treatment may counteract *KLHL21* loss observed in gastric cancer, which is known to enhance STAT3 activation and promote tumour progression [153]. Restoring *KLHL21* expression could improve chemotherapy response and inhibit the transition from metaplasia to dysplasia, offering a novel therapeutic strategy for gastric cancer management [153].

Furthermore, histone cluster genes, including *H2BC9*, showed significant upregulation (log2FC = 2.51), indicating changes in nucleosome assembly and chromatin structure [154]. These histones play critical roles in DNA replication, repair, and epigenetic regulation [155]. Their altered expression suggests chromatin remodelling in response to APB+Dox, which may contribute to replication stress, cell cycle arrest, or apoptosis during the S phase. In contrast, *SLX9*, an *RNA* processing factor involved in ribosome biogenesis, was strongly downregulated (log2FC = −2.51) [156,157]. As ribosome synthesis is tightly linked to cell growth and the G1/S transition, *SLX9* suppression likely impaired protein synthesis, leading to growth arrest and enhanced cytotoxicity [156,157]. Finally, several genes involved in DNA repair and genome stability, including *WRN* (log2FC = 1.84), *RRM2* (log2FC = 1.00), and *XRCC3* (log2FC = 0.72), were upregulated, reflecting a cellular attempt to mitigate DNA damage induced by treatment [158,159,160]. However, despite these adaptive responses, the extent of DNA damage and stress caused by APB+Dox may exceed the repair capacity, tipping the balance toward apoptosis and tumour regression.

### 2.4. Flow Cytometric Analyses of Apoptotic Profiles of Mono and Combination Therapies

**Figure 6:**
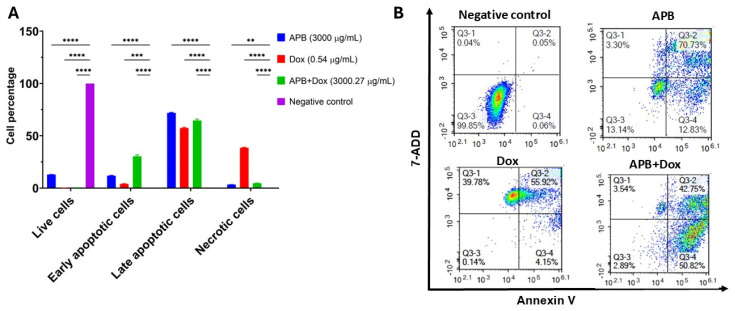
Flow cytometric assessment of the apoptotic profiles of the AGS gastric cancer cells after 24 h of treatment. (**A**) The live, early apoptotic, late apoptotic, and necrotic cell percentages after 24 h treatment with APB (3000 µg/mL), Dox (0.54 µg/mL), APB+Dox (3000.27 µg/mL), and control (n = 4). ** Indicates 0.01 < *p*-value < 0.05; *** indicates *p* < 0.001; **** indicates *p* < 0.0001 compared to the negative control. (**B**) Represented are the density plots of each drug treatment that is most representative of the average data from the flow cytometric analyses, with Q3-1 = necrotic cells, Q3-2 = late-stage apoptotic cells, Q3-3 = live cells, and Q3-4 = early-stage apoptotic cells.

Recognized as a fundamental barrier to cancer progression, apoptosis, a form of programmed cell death, eliminates potentially malignant cells from the body, making it a central target in the development of many modern anticancer therapies [123]. In this study, flow cytometry was used to distinguish between apoptotic and necrotic cell populations through the application of Annexin V and 7-AAD. Annexin V specifically detects phosphatidylserine, a phospholipid that becomes exposed on the outer leaflet of the plasma membrane during early apoptosis. In contrast, 7-AAD binds strongly to guanine–cytosine-rich regions of double-stranded DNA, allowing for the identification of necrotic cells. The results are classified into four groups: live cells, early-stage apoptotic cells, late-stage apoptotic cells, and necrotic cells. The effects of the monotreatments APB and Dox, along with the combination APB+Dox, were investigated using flow cytometric analysis, compared to the negative control (Figure 6).

APB (3000 μg/mL) treatment produced 71.91% late apoptotic cells and 11.81% early apoptotic cells (*p* < 0.0001), with low necrosis (3.32%) and 12.96% live cells (*p* < 0.01). Dox (0.54 μg/mL) treatment resulted in 57.40% late apoptotic cells, 3.95% early apoptotic cells (*p* < 0.0001), and a higher necrotic fraction (38.49%), with only 0.16% live cells. The APB+Dox combination (3000 μg/mL + 0.27 μg/mL) yielded 64.56% late apoptotic cells and 30.27% early apoptotic cells (*p* < 0.0001), with 4.64% necrotic cells and 0.53% live cells. The necrotic proportion in the combination group was notably lower than in the Dox-only group (38.49%) (Figure 6).

### 2.5. ROS Production in the AGS Cells After Treatment with Different Concentrations of APB, Dox, and APB+Dox

**Figure 7:**
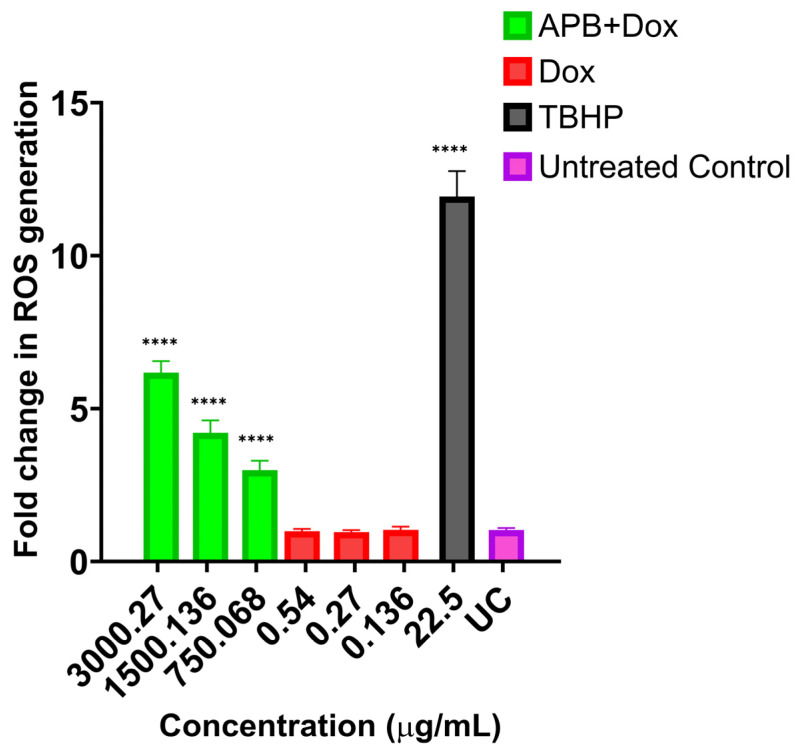
Fold change in ROS generation following treatment with various concentrations: 3000:0.27 μg/mL, 1500:0.14 μg/mL, and 750:0.07 μg/mL of APB+Dox. Additionally, Doxorubicin (0.54, 0.27, 0.14 μg/mL) and tert-Butyl hydroperoxide (TBHP) (22.5 μg/mL or 250 μM) are included for comparative purposes. The values are expressed as mean ± SD. **** indicates *p* < 0.0001 compared to untreated control.

ROS play a dual role in cancer biology, where their excessive accumulation, commonly referred to as oxidative stress, can contribute to both tumour initiation and progression [161]. Interestingly, manipulating ROS levels within cancer cells, either by promoting their accumulation to toxic levels or by disrupting the redox balance, has emerged as a promising approach in the development of anticancer therapies [161]. TBHP, used as a positive control for ROS induction, produced a significant increase in ROS levels (11.94-fold) compared to the untreated control (Figure 7). At 3000 μg/mL, APB alone induced a 6.14-fold increase in ROS relative to control. The combination of APB+Dox at the same concentration (3000 μg/mL + 0.27 μg/mL) resulted in a similar ROS increase (6.17-fold), indicating that Dox did not markedly alter APB’s pro-oxidative effect at this dose. APB combined with Dox at lower concentrations produced ROS levels of 2.99-fold. In contrast, Dox alone at comparable concentrations yielded lower ROS increases (0.99–1.04-fold).

## 3. Materials and Methods

### 3.1. Chemicals and Drug Preparation

All solvents used in this study were of analytical grade and procured from Sigma Aldrich (Castle Hill, NSW, Australia). Magnesium acetate (A), sodium propionate (P), sodium butyrate (B), and Dox were also obtained from Sigma Aldrich (Castle Hill, NSW, Australia). Additionally, all reagents were prepared following the standard procedures and protocols specified in the assay kits.

### 3.2. Cell Culture

The AGS gastric adenocarcinoma (CRL-1739, ATCC) and HS738.St/Int stomach intestinal (CRL-7869, ATCC) cell lines were sourced from the American Type Culture Collection (ATCC, Manassas, VA, USA). AGS cells were maintained in ATCC-formulated F-12K medium (Kaighn’s Modification of Ham’s F-12), supplemented with 2 mM L-glutamine, 1500 mg/L sodium bicarbonate, 10% foetal bovine serum (Bio-Strategy PTY, Campbellfield, VIC, Australia), and 1% penicillin–streptomycin (Sigma-Aldrich, Castle Hill, NSW, Australia). HS738.St/Int cells were cultured in ATCC-formulated DMEM (Dulbecco’s Modified Eagle Medium, ATCC, VA, USA). containing 4.5 g/L glucose, L-glutamine, sodium pyruvate, 10% foetal bovine serum, and 1% penicillin–streptomycin. Both cell lines were incubated at 37 °C in a humidified atmosphere of 5% CO_2_, with maintenance performed every 48–72 h to sustain confluent monolayers.

### 3.3. Cell Viability Assays

The cell viability of AGS cells after exposure to varying concentrations of SCFAs combinations (APB), Dox, and the combined treatment (APB+Dox) was evaluated using the Alamar Blue assay, following previously established protocols [162]. Briefly, 100 µL of AGS cells were seeded at a density of 10^5^ cells/mL into 96-well plates and allowed to adhere for 24 h. The cells were then treated with the test samples and incubated for 72 h. Dox at a concentration of 1 µM was also used as a positive control, while 0.1% DMSO served as a negative control across all plates. After the incubation period, the culture medium was removed, and 100 µL of a freshly prepared 0.1 mg/mL Alamar Blue solution was added to each well. The Alamar Blue solution was made by diluting a 1 mg/mL resazurin stock in PBS at a 1:10 ratio with serum-free media. Fluorescence measurements were obtained using a microplate spectrophotometer (BMG CLARIOstar, Mornington, VIC, Australia) with excitation and emission wavelengths of 555 nm and 595 nm, respectively. Each sample was tested in triplicate, and cell viability in the negative control group was normalized to 100%.

### 3.4. Synergy

Dox was combined with the SCFAs mixture APB at a 1:1 ratio to perform combination index (CI) analyses. The potential interactions between Dox and APB were evaluated using the CI model, with calculations conducted via CompuSyn version 2.0 software (Biosoft, El Cajon, CA, USA). The CI calculations were based on the median-effect equation, derived from the mass action law [163]. In this study, the APB+Dox combination was analysed using a six-point dose–response curve within the CI framework.

### 3.5. Liquid Chromatography–Mass Spectrometry, Label-Free Quantification Bottom-Up Proteomics

All reagents were purchased from Thermo Fisher Scientific (Waltham, MA, USA) unless stated otherwise. Proteomics studies were conducted following a recently established protocol. AGS gastric adenocarcinoma cells were cultured and treated in T75 flasks with APB, Dox, or their combination, APB+Dox, for 24 h. Post-treatment, cells were harvested, washed, and lysed in a sodium-deoxycholate-based buffer. Protein extraction involved sonication, centrifugation, and acetone precipitation. Protein pellets were reduced, alkylated, quantified, and digested overnight with trypsin. Peptides were desalted, dried, and resuspended in loading buffer before LC–MS analysis. Peptide separation was performed using nano-liquid chromatography (Ultimate 3000 HPLC, (Thermo Fisher Scientific, Waltham, MA, USA with a 75 μm × 45 cm C18 column and a gradient elution protocol. Mass spectrometry was conducted on a Q Exactive HF-X Orbitrap (Thermo Fisher Scientific, Waltham, MA, USA), in data-independent acquisition (DIA) mode, capturing full MS scans and sequential fragmentation across *m*/*z* windows. Raw data were processed using Spectronaut (v19) with the directDIA+ workflow against the 2023 UniProt human database. Parameters included trypsin digestion, dynamic modifications (oxidation, N-terminal acetylation), and fixed carbamidomethylation. False discovery rates for peptide-spectrum matches, peptides, and protein groups were set at 1%. Quantification was based on MS1 peak area, and differential abundance was assessed using unpaired t-tests. Data were deposited in PRIDE (PXD061824).

### 3.6. Flow Cytometry

The effects of APB, Dox, and APB+Dox on the apoptosis profiles of AGS adenocarcinoma cells were evaluated using an annexin V and 7-AAD-based apoptosis detection kit (#ab214663, Abcam, Melbourne, VIC, Australia) following established protocols [33,163]. Briefly, AGS gastric cancer cells (1 × 10^6^ cells/10 mL) were seeded in T75 flasks and incubated for 24 h at 37 °C with 5% CO_2_. Cells were then treated with APB (3000 µg/mL), doxorubicin (Dox, 0.54 µg/mL), their combination (APB+Dox), and just growth media (untreated control). After 24 h of treatment, apoptosis was assessed using the Annexin V-CF Blue/7-AAD staining kit (Abcam, #ab214663), following the manufacturer’s instructions. Cells were harvested, stained, and analysed via flow cytometry (Novocyte 3000, ACEA Biosciences, San Diego, CA, USA). Data were initially gated to exclude debris and doublets based on forward and side scatter properties. Cell populations were then classified according to Annexin V and 7-AAD fluorescence as follows: live cells (Annexin V^−^/7-AAD^−^), early apoptotic cells (Annexin V^+^/7-AAD^−^), late apoptotic cells (Annexin V^+^/7-AAD^+^), and necrotic cells (Annexin V^−^/7-AAD^+^). Data from three independent biological replicates per group were analysed using NovoExpress software (v1.5.0).

### 3.7. ROS Production Analysis

The impact of Dox, APB, and their combination (APB+Dox) on oxidative stress in AGS gastric adenocarcinoma cells was assessed using the H2DCFDA (2′,7′-dichlorofluorescein diacetate) Cellular Reactive Oxygen Species (ROS) Detection Assay Kit (#ab113851; Abcam, Melbourne, VIC, Australia) following a recently established protocol [164]. Briefly, AGS cells were seeded at a density of 2.5 × 10^5^ cells/mL in a 96-well plate and allowed to adhere overnight. To measure ROS levels, the cells were treated with 20 μM H2DCFDA for 45 min. After removing the dye solution, the cells were washed with 1x buffer and then exposed to 750, 1500, and 3000 µg/mL of SCFAs combination APB; 0.54 µg/mL (1 μM) of Dox, APB+Dox (1:1), and 250 μM of tert-butyl hydroperoxide (TBHP; positive control). The cells were incubated at 37 °C for 4 h and fluorescence was measured using a microplate spectrophotometer (BMG CLARIOstar, Mornington, VIC, Australia) at an excitation/emission wavelength of 485/535 nm. The fold change in ROS production was calculated relative to the untreated control, where cells were treated with buffer according to the manufacturer’s instructions.

### 3.8. Statistical Analysis

Data collection and analysis were carried out using Microsoft Excel (MS Office 2021) for data management and GraphPad Prism (version 9.0, San Diego, CA, USA) for statistical analysis and visualization. All experiments were conducted in triplicate, with results expressed as the mean ± standard deviation (SD). Statistical significance between mean values was evaluated using a two-way ANOVA, with a significance threshold set at *p* < 0.05. The IC50 values, representing the concentration of a drug required to inhibit cell growth by 50%, were calculated using GraphPad Prism 9.0. Non-linear regression analysis was also performed using the same software for IC50 determination. The designation n = 3 corresponds to the number of independent biological replicates included in the analyses.

## 4. Conclusions

The current study shows that dietary SCFAs, specifically acetate, propionate, and butyrate (APB), can work synergistically with Dox to enhance anticancer activity in AGS gastric cancer cells. Our findings demonstrate that the APB+Dox combination significantly enhances antiproliferative effects compared to either agent alone, as evidenced by a substantial reduction in IC_50_ values and a strong synergistic interaction confirmed by combination index analysis. Mechanistically, this synergy appears to be mediated through multiple pathways. The APB+Dox combination exerted a synergistic anticancer effect on AGS gastric cancer cells by modulating key pathways involved in cell survival, apoptosis, cell cycle regulation, immune evasion, and tumour microenvironment remodelling.

The proteomic analyses revealed that the combination treatment significantly downregulated tumour-promoting genes such as *HLA-F*, *TRIAP1*, *TMBIM6*, and *ENTPD8*, suggesting reduced mitochondrial integrity and immune escape, thereby enhancing apoptotic susceptibility. Simultaneously, upregulation of *SERPINE1*, *USP17L4*, and components of the TP53 network reflected increased genotoxic stress responses and proteostasis alterations. Network and pathway enrichment analyses highlighted suppression of CD44-centred signalling, epithelial–mesenchymal transition (EMT), and fibrogenesis-related genes, indicating impaired tumour plasticity and reduced metastatic potential. Canonical pathway analysis revealed pronounced activation of DNA damage checkpoints, oxidative stress responses, and cell cycle arrest, supporting the enhanced cytostatic and proapoptotic effects of APB+Dox. Apoptosis induction occurred via both intrinsic and extrinsic pathways, with increased expression of proapoptotic regulators (*TP53*, *BCL2L11*) and downregulation of survival-promoting genes (*MDK*, *TNFRSF10B*, *DCAF13*), tipping the balance toward programmed cell death. The combination also disrupted ribosome biogenesis and RNA processing, impairing protein synthesis and contributing to metabolic stress. Finally, modulation of haemostasis-related factors such as *MDK* and *TACSTD2* further inhibited angiogenesis and tumour-associated signalling. Altogether, this study demonstrates that APB enhances Dox efficacy by disrupting AGS cell homeostasis at multiple levels, supporting its potential role as a promising adjuvant strategy in gastric cancer therapy.

While these findings provide valuable mechanistic evidence for the synergistic anticancer effects of APB and Dox in gastric cancer cells, further discussion is warranted regarding their potential translation into real-world clinical settings. In particular, considerations such as physiological relevance of SCFA concentrations, feasibility of achieving therapeutic levels through diet or supplementation, interactions with standard chemotherapy regimens, patient heterogeneity, and safety profiles in vivo should be addressed. Importantly, the clinical implementation of SCFA-based adjuvant strategies also presents both challenges and opportunities. SCFAs are endogenously produced metabolites with favourable safety profiles, but their delivery alongside chemotherapy will require careful consideration of formulation, route of administration, and stability. Potential approaches may include dietary modulation, prebiotic supplementation to enhance endogenous SCFA production, or controlled-release formulations designed to achieve sustained and localised exposure. Addressing these practical aspects through preclinical animal studies and well-designed clinical trials will be essential to determine whether SCFA-based adjuvant strategies can meaningfully enhance therapeutic outcomes and reduce chemotherapy-associated toxicity in patients with gastric cancer.

## Figures and Tables

**Table 1:** Cell growth inhibition (%) against the AGS gastric adenocarcinoma and cell viability (%) of the Hs 738.St/Int normal intestine cell lines at different concentrations of APB combination, doxorubicin (Dox), combination APB+Dox, and combination for 72 h using the Alamar Blue assay (n = 3).

Conc. μg/mL	Cell viability (%) HS738.St/Int	Conc. μg/mL	Cell Growth Inhibition (%) of AGS Cels	Cell Viability (%) HS738.St/Int	Conc. μg/mL	Cell Growth Inhibition (%) of AGS Cels	Cell Viability (%) HS738.St/Int
	APB		Dox			APB+Dox	
3000	76.59 ± 8.56 ^a^	0.54	73.51 ± 5.16 ^a^	38.37 ± 7.01 ^a^	3000 + 0.27	103.46 ± 2.24 ^a^	64.12 ± 8.76 ^a^
1500	79.67 ± 8.16 ^a^	0.27	34.69 ± 2.96 ^b^	62.84 ± 11.53 ^b^	1500 +0.136	102.51 ± 9.05 ^a^	92.42 ± 10.66 ^b^
750	80.98 ± 9.19 ^a^	0.136	20.77 ± 7.29 ^c^	68.52 ± 7.51 ^b^	750 + 0.068	85.89 ± 8.58 ^b^	100.54 ± 8.51 ^b^
375	85.80 ± 13.04 ^a^	0.068	17.29 ± 7.96 ^c^	71.51 ± 7.81 ^b^	375 + 0.034	50.34 ± 8.49 ^c^	105.92 ± 11.80 ^b^
187.5	103.99 ± 10.54 ^b^	0.034	9.77 ± 6.90 ^c^	87.28 ± 9.25 ^c^	187.5 + 0.017	28.04 ± 7.40 ^d^	112.90 ± 11.32 ^b^
93.75	117.67 ± 13.09 ^b^	0.017	1.21 ± 3.84 ^c^	89.33 ± 11.72 ^c^	93.75 + 0.0085	27.29 ± 11.68 ^d^	114.72 ± 11.16 ^b^
**IC_50_**	**>3000**	**IC_50_**	**0.22 ± 0.04**	**>0.27**	**IC_50_**	**512.80 ± 18.37**	**>3000.27**

^a,b,c,d^ The different superscript values in the same column for each cell line indicate statistically significant difference (*p* < 0.05) compared to the highest concentration (3000 μg/mL).

## Data Availability

The raw and processed data have been deposited to the ProteomeXchange.Consortium via the PRoteomics IDEntifications (PRIDE) repository with the dataset identifier PXD061824.

## Supplementary Materials

The following supporting information can be downloaded at: https://www.mdpi.com/article/10.3390/ijms27010362/s1.
