# Supplementary material for: Postbiotics Combination Synergises the Antiproliferative Effects of Doxorubicin in Gastric Cancer Cells: A Cellular and Molecular Deep Dive
Source: Int J Mol Sci. 2025 Dec 29;27(1):362. doi: 10.3390/ijms27010362 (PMC12785482; doi:10.3390/ijms27010362)
Supplement: Supplementary file 1 [file ijms-27-00362-s001.zip › Supplementary Figures.pdf]

Postbiotics Combination Synergises the Antiproliferative Effects of Doxorubicin in Gastric Cancer Cells: A Cellular and Molecular Deep Dive

Radwa A. Eladwy [1,2,*], Mohamed Fares [3], Muhammad A. Alsherbiny [4,5], Dennis Chang [1], Chun-Guang Li [1] and Deep Jyoti Bhuyan [1,6,*]

1 NICM Health Research Institute, Western Sydney University, Penrith, NSW 2751, Australia; d.chang@westernsydney.edu.au (D.C.); c.li@westernsydney.edu.au (C.-G.L.)
2 Department of Pharmacology, Faculty of Pharmacy, Egyptian Russian University, Badr City 11829, Egypt
3 Sydney Pharmacy School, The University of Sydney, Sydney, NSW 2006, Australia; mohamed.metwaly@sydney.edu.au
4 Pharmacognosy Department, Faculty of Pharmacy, Cairo University, Cairo 11562, Egypt; m.ali@victorchang.edu.au
5 Freedman Foundation Metabolomics Facility, Victor Chang Cardiac Research Institute, Darlinghurst, NSW 2010, Australia
6 School of Science, Western Sydney University, Penrith, NSW 2751, Australia
* Correspondence: 22027531@student.westernsydney.edu.au (R.A.E.); d.bhuyan@westernsydney.edu.au (D.J.B.)

Academic Editors: Shu Yuan and Takuji Tanaka

Received: 5 December 2025
Revised: 20 December 2025
Accepted: 27 December 2025
Published: 29 December 2025

Copyright: © 2025 by the authors. Submitted for possible open access publication under the terms and conditions of the Creative Commons Attribution (CC BY) license.

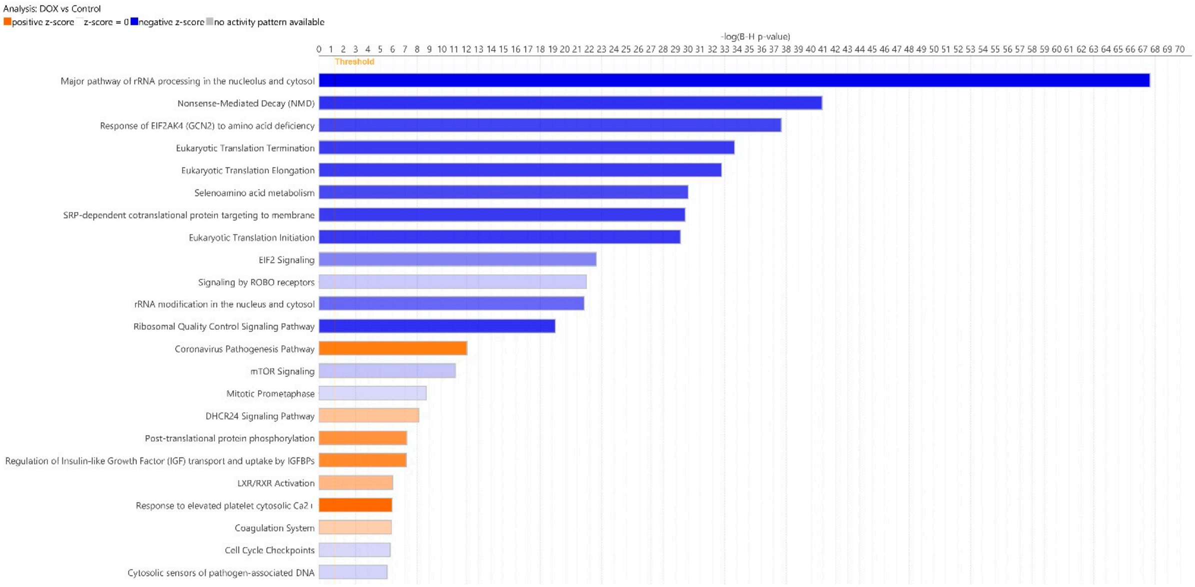

Figure S1: IPA enriched canonical pathways that identify the most significant signalling and metabolic pathways in the Dox group and predicts whether each pathway is activated (red) or inhibited (blue). Chart was filtered to include enriched term with -log BH-P value >1.3 and Abs z score >= 1

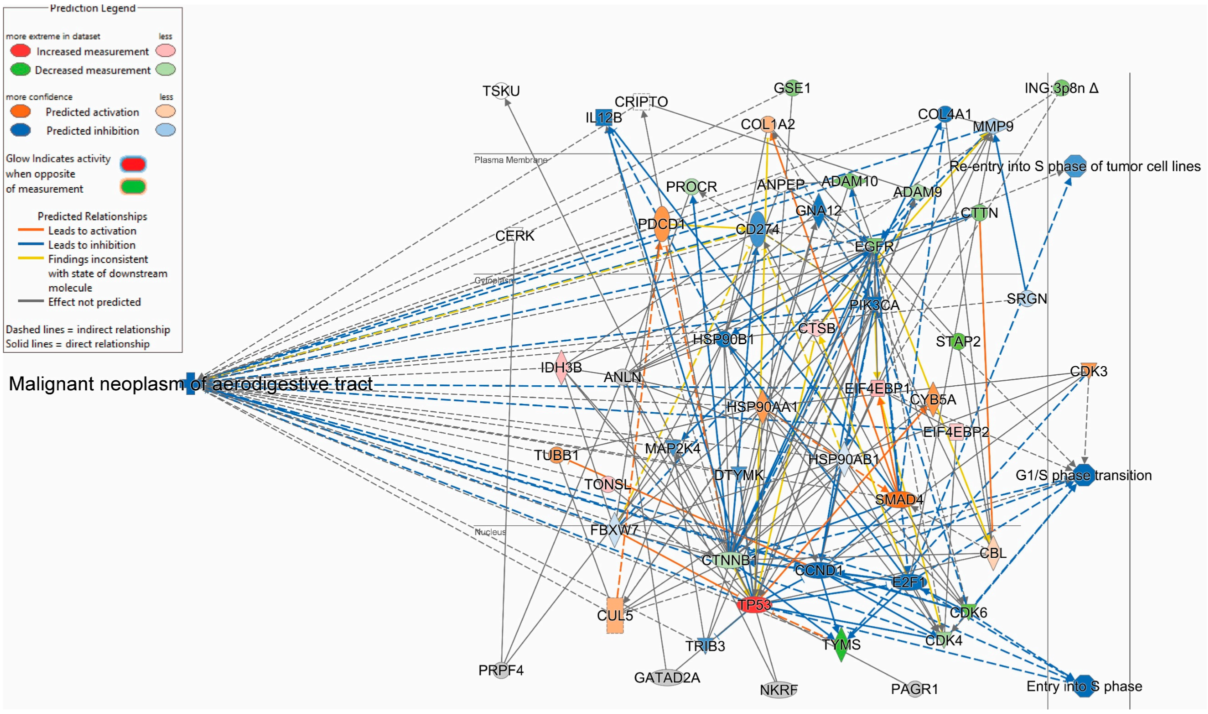

Figure S2. Machine learning-based prediction of APB+Dox's (vs control) Molecular Effects on malignant neoplasm of aerodigestive tract signalling pathway

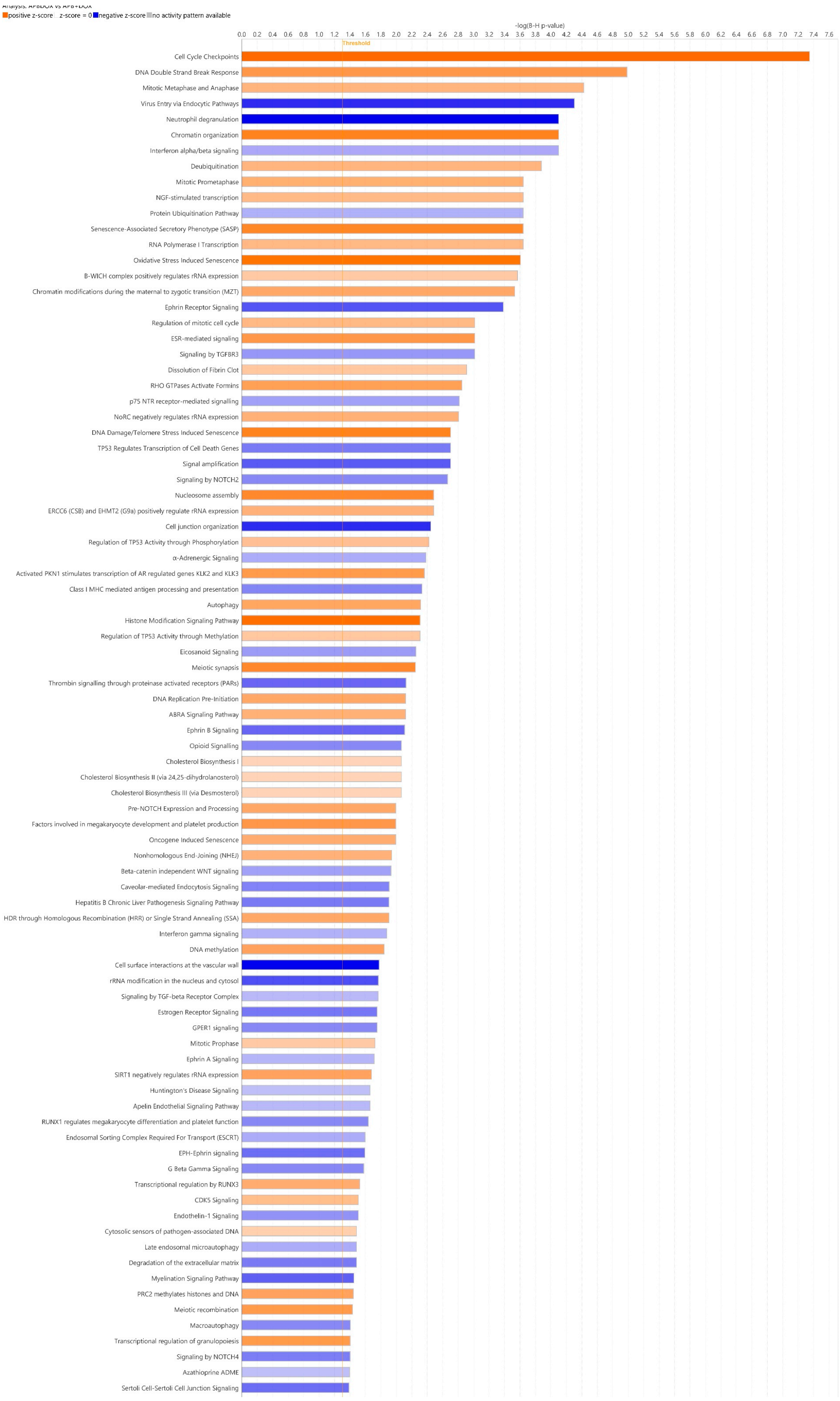

Figure S3: IPA enriched canonical pathways that identify the most significant signalling and metabolic pathways in the APB+Dox group and predicts whether each pathway is activated (red) or inhibited (blue). Chart was filtered to include enriched term with -log BH-P value >1.3 and Abs z score >= 1

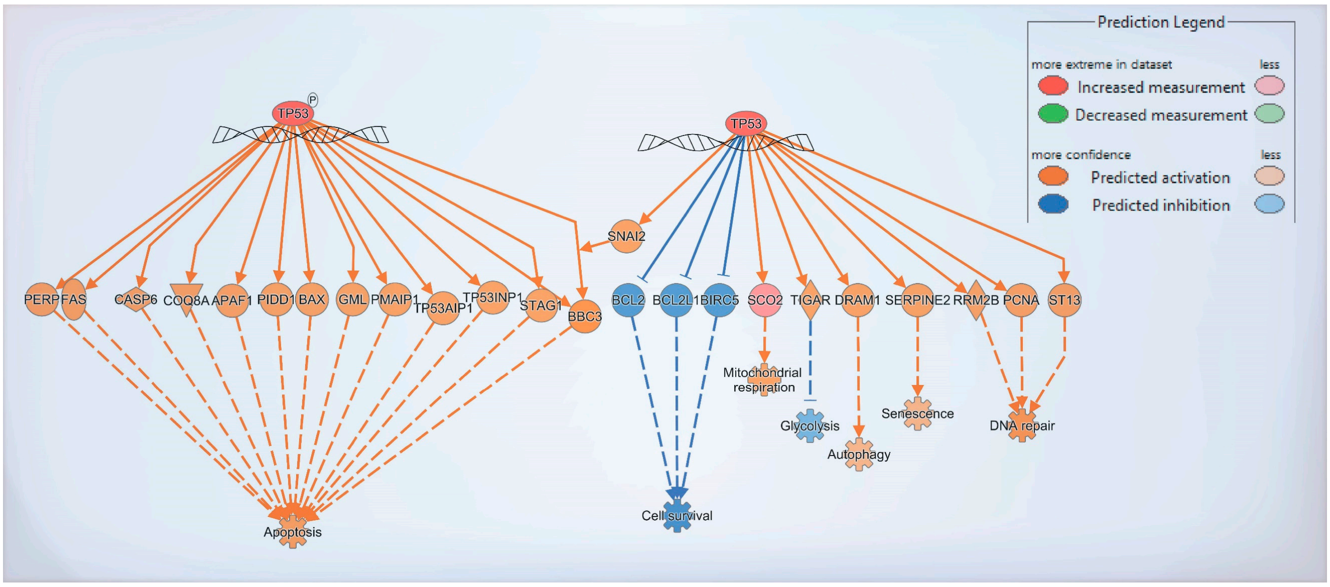

Figure S4. TP53-mediated regulatory network illustrating the predicted activation of apoptosis and autophagy pathways, along with the inhibition of cell survival pathways following APB+Dox combination treatment.

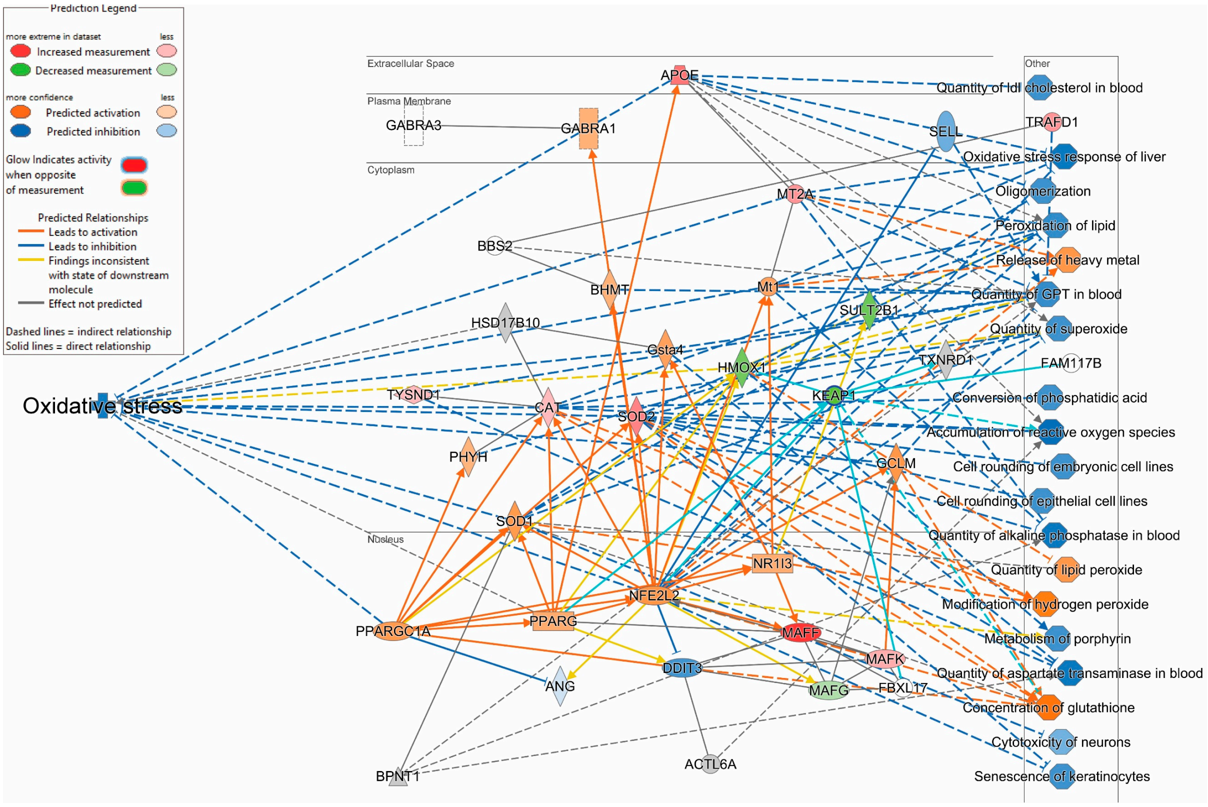

Figure S5. Machine learning-based prediction of APB+Dox's (vs APB and Dox individual treatments) molecular effects on malignant neoplasm of aerodigestive tract signalling pathway
